# Patient Satisfaction Among Complete and Partial Removable Denture Wearers: A Questionnaire Survey

**DOI:** 10.7759/cureus.117836

**Published:** 2026-10-13

**Authors:** Adrita R Chowdhury, Priya Bagal, Hardik Dholakiya, Sameer Chauhan, T R Manjunatha, Manisha Nagar

**Affiliations:** 1 Department of Prosthodontics, North Bengal Dental College and Hospital, Darjeeling, IND; 2 Department of Prosthodontics, Shaheed Kartar Singh Sarabha Dental College and Hospital, Sarabha, IND; 3 Department of Dentistry, Thundermist Health Center, Woonsocket, USA; 4 Department of Prosthodontics, K. M. Shah Dental College and Hospital, Sumandeep Vidyapeeth (Deemed to be University), Vadodara, IND; 5 Department of Prosthodontics, Baba Jaswant Singh Dental College, Hospital and Research Institute, Ludhiana, IND; 6 Department of Dentistry, Shree Rushabh Ajit Jain Medical and Charitable Trust, Thane, IND

**Keywords:** complete denture, oral health-related quality of life, partial, patient satisfaction, removable

## Abstract

Introduction

Patient satisfaction is an important outcome for evaluating the success of removable prosthodontic rehabilitation. However, satisfaction may differ according to the type of prosthesis and change as patients adapt to their dentures. This study aimed to compare patient satisfaction among complete and removable partial denture (RPD) wearers at prosthesis delivery and after three months of use and to evaluate changes in satisfaction over this period.

Materials and methods

A prospective questionnaire-based study was conducted in the Department of Prosthodontics in a hospital in India in 2024. In total, 780 patients were included, comprising 455 complete removable denture and 325 RPD wearers. A 10-item patient-satisfaction questionnaire assessing the functional, comfort-related, esthetic, and psychosocial domains was administered at prosthesis delivery and after three months. Data were analyzed using the Mann-Whitney U test, Wilcoxon signed-rank test, and chi-square or Fisher's exact test, as appropriate.

Results

At prosthesis delivery, RPD wearers reported higher total satisfaction than complete removable denture wearers, with median scores of 37 and 34, respectively (p<0.001). After three months, satisfaction increased to 41 and 40, respectively, and the between-group difference was no longer significant (p=0.081). The improvement in satisfaction was significant in both groups (p<0.001) and was greater among complete removable denture wearers. The questionnaire demonstrated excellent internal consistency, with Cronbach's α of 0.91 at delivery and 0.94 after three months.

Conclusion

Patient satisfaction improved significantly during the first three months of removable denture use. Although RPD wearers showed greater initial satisfaction, the difference largely diminished following adaptation, highlighting the importance of follow-up and patient-centered prosthodontic care.

## Introduction

Removable dentures remain an important treatment option for the rehabilitation of patients with partial or complete tooth loss, particularly when fixed or implant-supported treatment is not feasible because of anatomical, financial, or patient-related considerations [1]. Complete and removable partial dentures (RPDs) differ considerably in design, support, retention, stability, and functional demands. Consequently, patients receiving these prostheses may experience different levels of comfort and satisfaction during the initial period of use [2]. Although the clinical assessment of prosthetic fit and function is important, the success of removable prosthodontic treatment cannot be determined solely by clinician-based parameters. Patient satisfaction is an essential patient-reported measure that reflects an individual's perception of comfort, function, appearance, and psychosocial acceptability of the prosthesis [2,3].

Patient satisfaction with removable dentures is multidimensional and may be influenced by several factors, including overall fit and comfort, ability to chew and speak, retention and stability, appearance, ease of insertion and removal, oral tissue comfort, cleaning and maintenance, and confidence in social situations [2-4]. These factors may be particularly relevant immediately following prosthesis delivery when patients adapt to a new prosthesis. With continued use, neuromuscular adaptation and increasing familiarity with dentures may improve functional performance and overall satisfaction [5]. Therefore, evaluating satisfaction only at the time of prosthesis delivery may not adequately reflect a patient's eventual experience with the prosthesis.

A comparison between complete removable denture wearers and RPD wearers is also clinically relevant because the extent of tooth loss and the type of prosthetic support may influence initial satisfaction and subsequent adaptation [6]. Understanding whether the differences between the two groups persist after a period of prosthesis use can provide useful information for patient counselling and prosthodontic treatment planning. A structured patient-satisfaction questionnaire can facilitate a systematic assessment of subjective outcomes across multiple domains and allow changes in satisfaction to be evaluated over time [7]. Therefore, the aim of this study was to compare patient satisfaction among complete removable denture and RPD wearers at the time of prosthesis delivery and after three months of use and to evaluate the change in satisfaction within and between the two groups. The objectives were to assess satisfaction across different domains of removable denture use, compare overall and individual-domain satisfaction between complete removable denture wearers and RPD wearers at both assessment points, determine changes in total satisfaction after three months of prosthesis use, and assess the internal consistency of the patient-satisfaction questionnaire.

## Materials and methods

Study design and setting

This prospective questionnaire-based study was conducted in the Department of Prosthodontics, Baba Jaswant Singh Dental College, Hospital and Research Institute, Ludhiana, India. The study was conducted from March to July 2024. This study was approved by the Institutional Ethics Committee of the college (BJSDCH&RI/IEC/2024/02/26). Written informed consent was obtained from all participants prior to their inclusion in the study. A total of 780 eligible participants were enrolled at prosthesis delivery and classified into complete removable denture (n=455) and removable partial denture (n=325) groups. Patient satisfaction was assessed at prosthesis delivery and again after three months of denture use. The participant flow from eligibility assessment through follow-up and final analysis is summarized in Figure 1.

**Figure 1:**
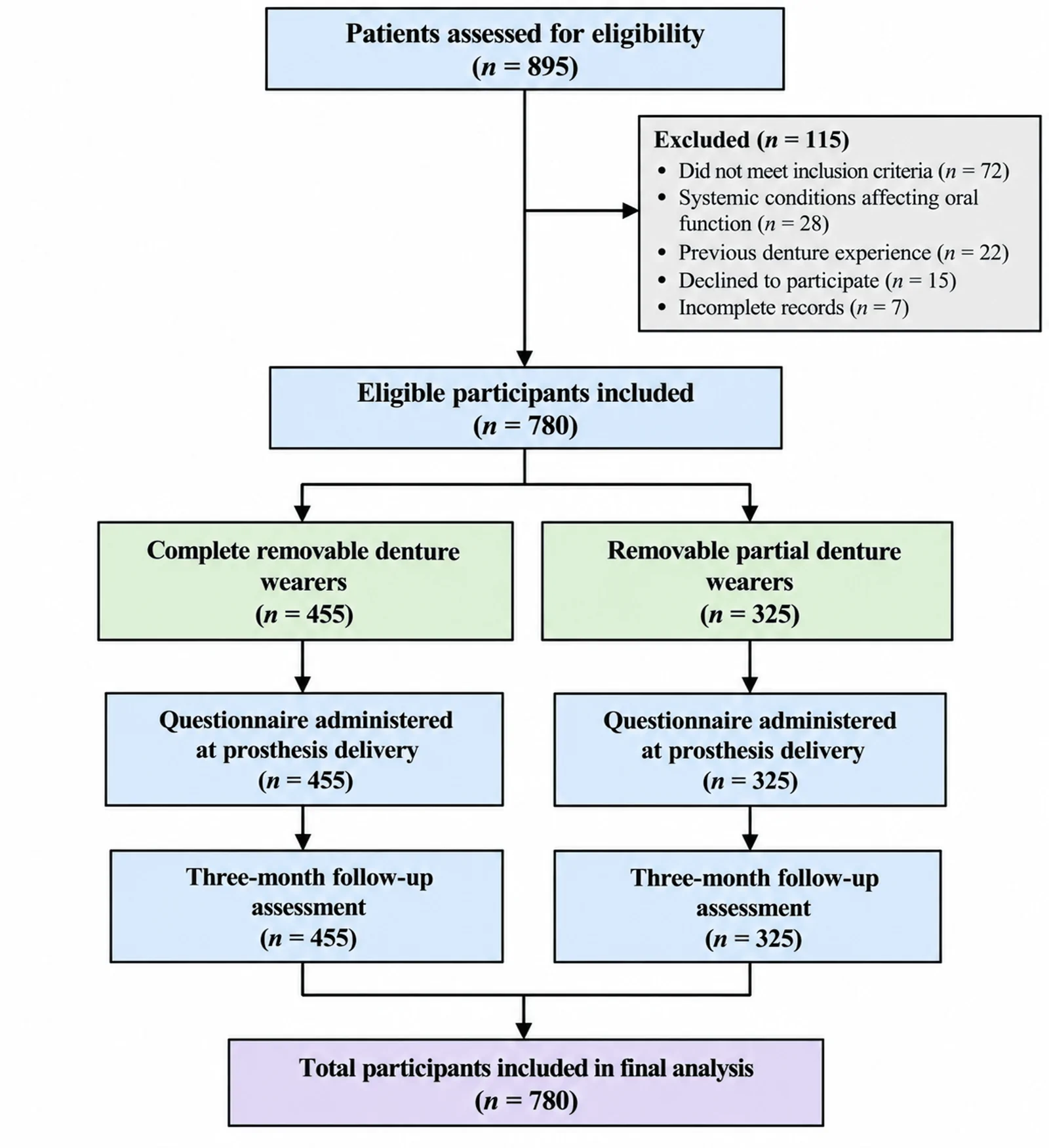
Flowchart of participant recruitment, eligibility, follow-up, and final analysis. The participant flowchart was created using Canva (Canva Pty Ltd, Sydney, NSW, Australia). No artificial intelligence (AI)-based features were used in the creation or editing of the figure.

Sample size and statistical power

A total enumeration sampling strategy was used, and all eligible patients available during the study period were included. Consequently, no prospective sample size calculation was used to determine recruitment because the study included the entire accessible eligible population. The final analytical sample comprised 780 (91.7%) patients, including 455 (60.7%) complete removable denture wearers and 325 (43.3%) RPD wearers. A post hoc power calculation using G*Power software version 3.1.9.7 (Heinrich Heine University Düsseldorf, Düsseldorf, Germany) based on the observed between-group effect for the change in the total satisfaction score (r=0.31; approximately Cohen's d=0.65) indicated a statistical power greater than 99% for a two-sided comparison at α=0.05. The study population comprised patients attending the department for prosthetic rehabilitation.

Development of the questionnaire

A structured, self-administered patient satisfaction questionnaire was developed specifically for the present study to assess patients' perceptions of their removable prostheses. The questionnaire was prepared in consultation with four prosthodontists from different dental institutions (ARC, PB, SC, and MN), with the objective of incorporating a broad range of clinically relevant and patient-centered aspects of removable denture satisfaction. The items were developed to cover major domains that may influence patients' experience with removable dentures, including prosthetic fit and comfort, appearance, mastication, speech, retention and stability, insertion and removal, oral tissue comfort, cleaning and maintenance, social confidence, and overall satisfaction.

The final questionnaire consisted of two sections (see Appendix, Table 8). The first section recorded demographic and patient-related information, including age, sex, educational level, occupation, and the type of removable prosthesis received. The second section consisted of 10 questions assessing satisfaction with different aspects of removable dentures. The satisfaction items evaluated the overall fit and comfort, appearance and smile, ability to chew and eat, speech clarity, retention and stability, ease of insertion and removal, oral tissue comfort, cleaning and maintenance, social confidence, and overall satisfaction.

Content validity

The content validity of the questionnaire was established through expert evaluation by four prosthodontists involved in its development. The experts independently reviewed the questionnaire items for their relevance, clarity, comprehensiveness, and appropriateness for assessing patient satisfaction with complete removable dentures and RPDs. The items were discussed and refined where necessary to ensure that the questionnaire adequately represented the principal domains of removable denture satisfaction and that the wording was understandable to the target patient population. The final version was prepared by incorporating recommendations from an expert panel.

Pilot testing and reliability assessment

Before commencement of the main study, the questionnaire was pilot-tested among a small group of 20 patients representative of the study population to assess its comprehensibility, acceptability, sequence of questions, and approximate time required for completion. The participants were asked to identify any questions that were unclear, ambiguous, repetitive, or difficult to understand. Based on the feedback obtained during pilot testing, the necessary linguistic and structural modifications were made before the questionnaire was used in the main study. The 20 patients included in the pilot test were excluded from the final study sample or subsequent statistical analysis. The internal consistency of the 10-item satisfaction questionnaire was assessed using Cronbach's alpha at two assessment points.

Eligibility criteria

Patients attending the Department of Prosthodontics for rehabilitation with a complete removable denture or RPD during the study period were included in the study. Eligibility criteria are summarized in Table 1.

**Table 1:** Inclusion and exclusion criteria. RPD: removable partial denture.

Inclusion criteria	Exclusion criteria
Patients attending the Department of Prosthodontics for rehabilitation with a complete removable denture or RPD during the study period.	Patients who were unable to understand or complete the questionnaire.
Patients who received the respective removable prostheses (complete removable denture or RPD).	Patients who did not provide the required information.
Patients assessed at the time of prosthesis delivery and after 3 month follow-up.	Patients who were unavailable for the three-month follow-up.

Clinical protocol

All prostheses were fabricated according to standardized clinical and laboratory protocols by the Department of Prosthodontics. Complete dentures were fabricated as conventional definitive prostheses for completely edentulous patients, whereas removable partial dentures were provided for partially edentulous patients according to the distribution of remaining teeth and the patient's clinical requirements. The prosthetic procedures included preliminary and definitive impressions, jaw-relation recording, tooth arrangement, try-in, processing, insertion, and necessary occlusal adjustments. Following insertion, patients received standardized post-insertion instructions regarding denture hygiene, use, and adaptation. Any required minor adjustments during follow-up were performed as part of routine prosthodontic care.

All prostheses were clinically evaluated at the time of insertion for fit, retention, stability, occlusion, border extension, and patient comfort. During the three-month follow-up period, patients were advised to return to the Department of Prosthodontics if they experienced pain, discomfort, instability, difficulty in mastication, speech-related problems, or any other prosthesis-related concern. Post-insertion adjustments were performed by the treating prosthodontist whenever clinically indicated and included pressure-spot relief, occlusal adjustment, correction of border extension, clasp adjustment for removable partial dentures, and minor denture-base adjustments, as appropriate. All adjustment procedures were documented in the patient's clinical record. No replacement or major modification of the prostheses was performed during the three-month follow-up period unless clinically indicated.

Questionnaire administration and assessment

The questionnaire was administered to participants at two predefined assessment points: immediately following prosthesis delivery and three months after prosthesis use. Participants were provided with standardized instructions and informed that there were no right or wrong answers. They were requested to answer each question according to their personal experience with removable dentures. The responses were treated as confidential and used exclusively for research purposes.

Each of the 10 satisfaction questions was scored on a five-point Likert scale ranging from 1 to 5, where 1 indicated “very dissatisfied,” 2 indicated “dissatisfied,” 3 indicated “neither satisfied nor dissatisfied,” 4 indicated “satisfied,” and 5 indicated “very satisfied.” The total satisfaction score was calculated by summing the scores for all 10 items. Thus, the minimum possible score was 10, and the maximum possible score was 50. Higher scores indicate greater patient satisfaction with the removable prosthesis. Based on a predefined scoring system (eight unit score per category), total scores of 10-17 represented very low satisfaction, 18-25 represented low satisfaction, 26-33 represented moderate satisfaction, 34-41 represented high satisfaction, and 42-50 represented very high satisfaction.

Data collection and blinding

The completed questionnaires were collected at two assessment points, and the responses were entered into the study database. To minimize the possibility of an investigator-related influence during statistical evaluation, the dataset provided for statistical analysis was anonymized and blinded with respect to participant identifiers and group-related identifying information. An independent statistician who was not involved in participant recruitment, questionnaire administration, or clinical assessment performed the statistical analysis. An independent analyst received the blinded dataset and performed statistical analyses according to the predefined analytical plan.

Statistical analysis

Statistical analyses were performed using IBM SPSS Statistics (version 25.0; IBM Corp., Armonk, NY, USA). The variables were assessed for normality using the Shapiro-Wilk test. As the satisfaction scores were not normally distributed, continuous data were summarized using median and interquartile range (IQR). The Mann-Whitney U test was used to compare the satisfaction scores between the complete removable denture and RPD groups. Changes in satisfaction scores from prosthesis delivery to three months within each group were evaluated using the Wilcoxon signed-rank test. Categorical variables were compared using Pearson's chi-square test or Fisher's exact test, as appropriate. The effect sizes were reported where appropriate, including Cramer's V for categorical comparisons and r for nonparametric comparisons. Statistical significance was set at p<0.05.

## Results

A total of 780 participants were included in the analysis, comprising 455 (58.3%) complete removable denture and 325 (41.7%) RPD users. Significant differences were observed between the groups in terms of age distribution (p<0.001), sex (p=0.046), and education (p = 0.002), whereas occupation did not differ significantly (p=0.873) (Table 2).

**Table 2:** Demographic characteristics of the study participants. Data are presented as frequency and percentage, categorical variables were compared using Pearson's chi-square test, effect size was expressed as Cramer's V, and *p<0.05 was considered statistically significant. df: degree of freedom.

Characteristic		Complete removable denture (n=455)	Removable partial denture (n=325)	Total (N=780)	Chi-square value (df)	p-value	Effect size
Age group, n (%)	41–50 years	20 (4.4)	65 (20.0)	85 (10.9)	87.75 (2)	< 0.001*	Cramer's V = 0.335
	51–60 years	150 (33.0)	155 (47.7)	305 (39.1)			
	≥60 years	285 (62.6)	105 (32.3)	390 (50.0)			
Sex, n (%)	Male	190 (41.8)	160 (49.2)	350 (44.9)	3.98 (1)	0.046*	Cramer's V = 0.071
	Female	265 (58.2)	165 (50.8)	430 (55.1)			
Education, n (%)	No formal education	80 (17.6)	35 (10.8)	115 (14.7)	14.33 (3)	0.002*	Cramer's V = 0.136
	Primary	190 (41.8)	125 (38.5)	315 (40.4)			
	Secondary	145 (31.9)	115 (35.4)	260 (33.3)			
	Graduate/postgraduate	40 (8.8)	50 (15.4)	90 (11.5)			
Occupation, n (%)	Employed	50 (11.0)	35 (10.8)	85 (10.9)	0.70 (3)	0.873	Cramer's V = 0.030
	Self-employed	110 (24.2)	80 (24.6)	190 (24.4)			
	Homemaker	95 (20.9)	75 (23.1)	170 (21.8)			
	Retired/unemployed	200 (44.0)	135 (41.5)	335 (42.9)			

At prosthesis delivery, the distribution of satisfaction scores was significantly higher among RPD wearers across all 10 domains. The greatest differences were observed for overall fit and comfort, chewing ability, and overall satisfaction, with small to moderate effect sizes (Table 3).

**Table 3:** Patient satisfaction across individual domains at prosthesis delivery. Data are presented as median (interquartile range), groups were compared using the Mann–Whitney U test, effect size was expressed as r, *p<0.05 was considered statistically significant. IQR: Interquartile range.

Satisfaction item	Complete removable denture (n=455)	Removable partial denture (n=325)	Test value (U)	p-value	Effect size (r)
	Median (IQR)	Median (IQR)			
S1. Overall fit and comfort	3 (3-4)	4 (3-4)	59,210	< 0.001*	0.24
S2. Appearance and smile	4 (3-4)	4 (4-5)	67,430	0.003*	0.11
S3. Ability to chew/eat	3 (3-4)	4 (3-4)	60,185	< 0.001*	0.21
S4. Speech clarity	3 (3-4)	4 (3-4)	65,210	0.001*	0.12
S5. Retention and stability	3 (3-4)	4 (3-4)	61,940	<0.001*	0.19
S6. Ease of insertion/removal	4 (3-4)	4 (4-5)	67,820	0.005*	0.10
S7. Oral-tissue comfort	3 (3-4)	4 (3-4)	63,180	<0.001*	0.17
S8. Cleaning/maintenance	4 (3-4)	4 (4-5)	69,050	0.018*	0.08
S9. Social confidence	3 (3-4)	4 (3-4)	64,020	<0.001*	0.16
S10. Overall satisfaction	3 (3-4)	4 (3-4)	60,540	<0.001*	0.23

After three months, satisfaction was largely comparable between the two groups. Significant differences persisted in fit and comfort (p=0.018), chewing ability (p=0.041), retention and stability (p=0.031), and oral tissue comfort (p=0.048), whereas the remaining domains showed no significant differences (Table 4).

**Table 4:** Patient satisfaction across individual domains after three months of prosthesis use. Data are presented as median (interquartile range), groups were compared using the Mann–Whitney U test, effect size was expressed as r, *p<0.05 was considered statistically significant. IQR: Interquartile range.

Satisfaction item	Complete denture (n=455)	Removable partial denture (n=325)	Test value (U)	p-value	Effect size (r)
	Median (IQR)	Median (IQR)			
S1. Overall fit and comfort	4 (3-5)	4 (4-5)	70,820	0.018*	0.08
S2. Appearance and smile	4 (4-5)	4 (4-5)	73,240	0.214	0.04
S3. Ability to chew/eat	4 (3-5)	4 (4-5)	71,190	0.041*	0.07
S4. Speech clarity	4 (3-5)	4 (4-5)	71,940	0.072	0.06
S5. Retention and stability	4 (3-5)	4 (4-5)	70,940	0.031*	0.08
S6. Ease of insertion/removal	4 (4-5)	4 (4-5)	72,980	0.184	0.05
S7. Oral-tissue comfort	4 (3-5)	4 (4-5)	71,280	0.048*	0.07
S8. Cleaning/maintenance	4 (4-5)	4 (4-5)	72,440	0.126	0.05
S9. Social confidence	4 (3-5)	4 (4-5)	71,760	0.061	0.07
S10. Overall satisfaction	4 (3-5)	4 (4-5)	72,090	0.109	0.06

Total satisfaction increased significantly in both groups from delivery to three months. In complete denture wearers, the median score increased from 34 (31-38) to 40 (35-44), while in RPD wearers it increased from 37 (34-40) to 41 (37-45); both changes were significant (p < 0.001) with large effect sizes (Table 5).

**Table 5:** Change in total patient satisfaction score from prosthesis delivery to three months. Data are presented as median (interquartile range), changes in satisfaction scores within groups were assessed using the Wilcoxon signed-rank test, effect size was expressed as r, *p<0.05 was considered statistically significant. IQR: Interquartile range.

Group	At delivery, median (IQR)	At three months, median (IQR)	Wilcoxon test	p-value	Effect size
Complete removable denture (n=455)	34 (31-38)	40 (35-44)	-18.62	<0.001*	0.87
Removable partial denture (n=325)	37 (34-40)	41 (37-45)	-13.94	<0.001*	0.77
Overall (N=780)	35 (32-39)	40 (36-44)	-22.96	<0.001*	0.82

RPD wearers had significantly higher satisfaction at delivery than complete removable denture wearers (p<0.001). However, this difference was no longer significant after three months (p=0.081). The improvement in satisfaction was significantly greater among complete removable denture wearers, with a median change of 6 (3-7) versus 4 (2-6) in RPD wearers (p<0.001) (Table 6).

**Table 6:** Comparison of total patient satisfaction scores between complete and removable partial denture wearers. Data are presented as median (interquartile range), groups were compared using the Mann–Whitney U test, effect size was expressed as r, *p<0.05 was considered statistically significant. IQR: Interquartile range.

Time point	Complete removable denture (n=455), median (IQR)	Removable partial denture (n=325), median (IQR)	Mann–Whitney U	p-value	Effect size (r)
At delivery	34 (31-38)	37 (34-40)	51,820	<0.001*	0.34
After three months	40 (35-44)	41 (37-45)	70,460	0.081	0.06
Change in score (T1-T0)	6 (3-7)	4 (2-6)	52,910	<0.001*	0.31

The distribution of satisfaction categories showed a clear shift toward higher satisfaction after three months of prosthesis use in both groups. The proportion of patients with low satisfaction decreased following the three-month follow-up, whereas the proportion reporting high and very high satisfaction increased. This shift was evident among both complete and RPD wearers and was consistent with the improvement observed in the total satisfaction scores (Figure 2).

**Figure 2:**
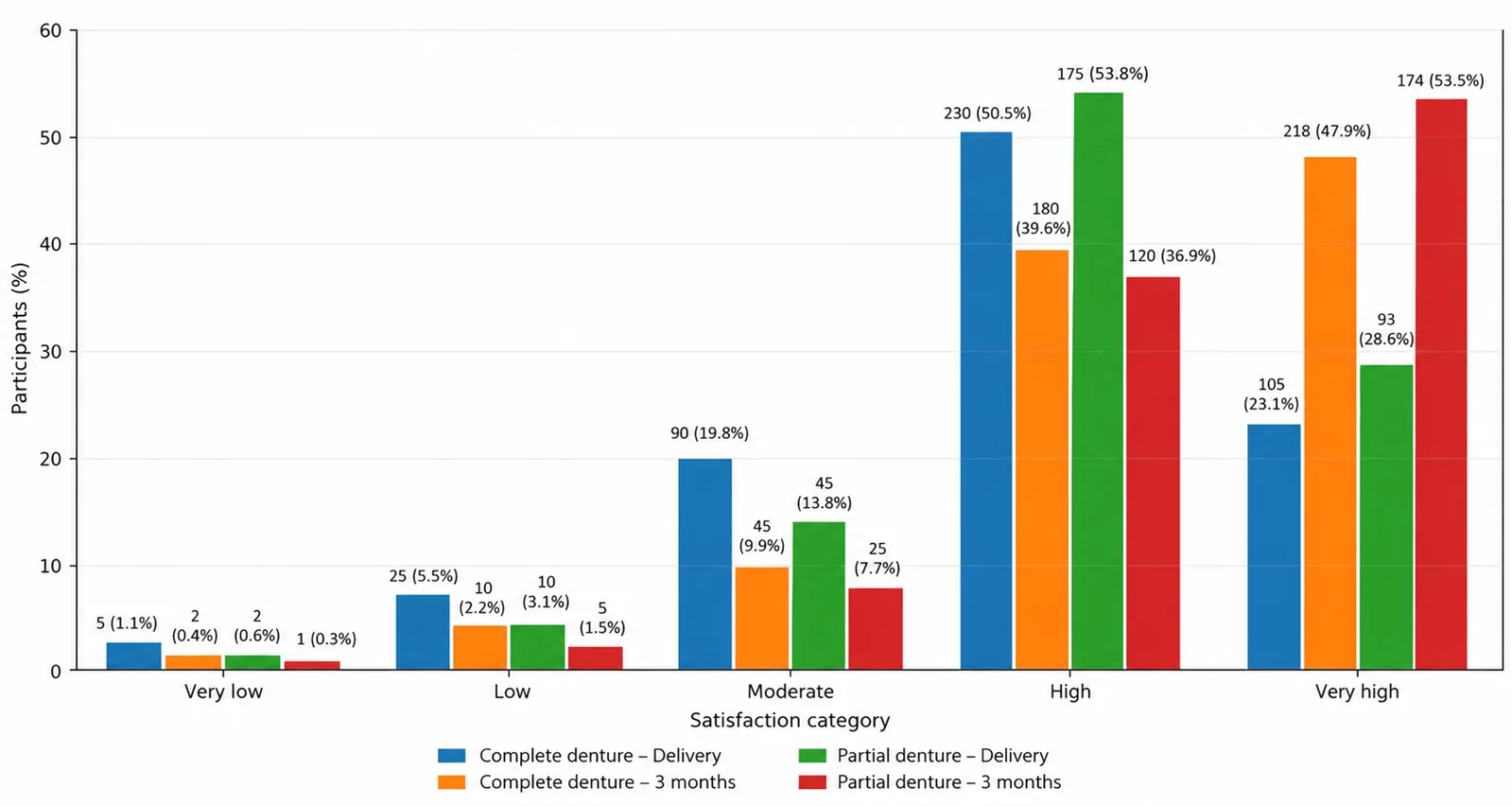
Distribution of patient satisfaction categories at prosthesis delivery and after three months of prosthesis use. Data are presented as frequency and percentage, satisfaction categories were based on the total score obtained from the 10-item patient-satisfaction questionnaire, higher categories indicate greater patient satisfaction, CD: complete removable denture, PD: removable partial denture.

The questionnaire demonstrated excellent internal consistency, with Cronbach's α of 0.91 at delivery, 0.94 at three months, and 0.93 overall (Table 7).

**Table 7:** Internal consistency of the patient-satisfaction questionnaire. Data are presented as the median inter-item correlation and Cronbach's alpha, internal consistency was assessed using Cronbach's alpha, Cronbach's alpha values ≥0.90 were considered indicative of excellent internal consistency.

Assessment	Number of items	Median inter-item correlation	Cronbach's α	Interpretation
Prosthesis delivery	10	0.48	0.91	Excellent
Three months	10	0.56	0.94	Excellent
Overall	10	0.52	0.93	Excellent

## Discussion

The present study evaluated patient satisfaction among complete removable denture and RPD wearers at prosthesis delivery and after three months of use. The findings indicated that the type of removable prosthesis was associated with differences in satisfaction at the initial assessment, but these differences became substantially less pronounced following the period of prosthesis use. Overall, satisfaction improved significantly over the follow-up period in both groups, suggesting that adaptation to and familiarity with the prosthesis may play an important role in early patient experience.

At the time of prosthesis delivery, RPD wearers demonstrated higher satisfaction across the assessed domains. This finding may be related to the greater preservation of natural teeth in partial denture wearers, which can provide additional support, stability, proprioception, and more familiar masticatory function [8]. In contrast, complete denture wearers must adapt to a prosthesis that replaces the entire dentition and relies predominantly on underlying oral tissues and denture-bearing structures for support and retention. Consequently, initial difficulties related to fit, stability, chewing, speech, and oral comfort may be more apparent among complete denture wearers [9]. Therefore, the observed differences across multiple satisfaction domains emphasize that the patient's initial perception of a newly delivered prosthesis may be influenced by the extent of edentulism and nature of prosthetic support [10].

After three months of prosthesis use, satisfaction was broadly comparable between the two groups. Although some differences persisted in specific domains, several aspects of satisfaction, including appearance, speech, insertion and removal, cleaning, social confidence, and overall satisfaction, did not differ significantly between the complete and partial denture wearers. This pattern is clinically meaningful because it suggests that the initial disadvantages experienced by complete denture wearers may diminish with continued use. Familiarity with the prosthesis, improvement in neuromuscular coordination, adaptation to changes in mastication and speech, and increased confidence in daily use may contribute to this improvement [5,11]. The reduction in between-group differences also supports the importance of evaluating prosthetic outcomes beyond the immediate postdelivery period.

An important finding was the significant improvement in overall satisfaction within both groups during the three-month follow-up period. This improvement was particularly pronounced among complete denture wearers, who demonstrated a greater change in satisfaction than partial denture wearers. This may reflect a greater scope for improvement among patients who initially experience greater difficulty adapting to a new complete denture. Similar findings were reported by Celebic et al. [6] and Gjengedal et al. [12]. These findings highlight that immediate post-insertion satisfaction should not necessarily be interpreted as the final outcome of treatment. Adjustment and appropriate follow-up may allow patients to overcome early functional and psychological challenges.

Previous studies have also compared the outcomes between complete and partial denture wearers. Nekouei et al. [13] reported that complete denture wearers experienced poorer oral health-related quality of life than partial denture wearers, suggesting that the extent of tooth loss and the type of prosthetic rehabilitation may influence patients’ perceived oral health outcomes. Similarly, Campos Sugio et al. [14] found that, although removable dentures improved oral rehabilitation, complete denture wearers showed greater limitations in masticatory efficiency than partial denture wearers, while both groups continued to experience some functional limitations.

De Kok et al. [15] reported that satisfaction and quality of life among removable partial denture wearers may be associated with factors such as age, number of occluding dental units replaced, replacement of anterior teeth, and nature of the opposing arch, although the evidence was not consistent across studies. These findings are broadly consistent with those of the present study, in which partial denture wearers demonstrated better satisfaction during the initial assessment, particularly in functional domains such as chewing, fit, and stability. However, the present findings further suggest that these initial differences may diminish with continued use and adaptation of prostheses. Chakaipa et al. [16] reported that patients receiving removable complete dentures valued accessibility, affordability, empathetic care, continuity, and effective communication, whereas delays and fragmented care negatively affected their experience.

Reliability analysis further supports the usefulness of the questionnaire in evaluating patient satisfaction. The instrument demonstrated excellent internal consistency at both assessment points, indicating that it showed good coherence in measuring the overall construct of removable denture satisfaction. The questionnaire incorporated several clinically relevant domains, rather than relying solely on a single global satisfaction question, thereby providing a broader assessment of the patient's experience.

From a clinical perspective, these findings emphasize the importance of patient counselling and structured follow-up after denture delivery. Patients with complete dentures may require particular attention during the early adaptation period, including the reinforcement of denture-wearing instructions, dietary advice, hygiene guidance, and management of discomfort or instability. Clinicians should also recognize that patient satisfaction may improve substantially over time and should encourage appropriate follow-up rather than considering early dissatisfaction as an indication of treatment failure. Assessment of patient-reported outcomes can complement conventional clinical evaluations and help identify domains that require additional prosthetic adjustment or patient education.

Limitations and future recommendations

This study has several limitations. First, it was conducted at a single dental institution, which may limit the generalizability of the findings to other patient populations and clinical settings. Second, the complete and partial denture groups differed significantly in baseline demographic characteristics, including age, sex, and educational level. As the study involved naturally occurring clinical groups and was not designed as a randomized or matched comparison, these differences may have influenced the observed between-group satisfaction scores. Therefore, the findings should be interpreted as unadjusted associations rather than causal effects attributable solely to denture type. Third, detailed prosthetic characteristics, including arch distribution, Kennedy classification of partially edentulous arches, and previous denture-wearing experience, were not prospectively recorded and therefore could not be incorporated into the analysis. These factors may influence patient satisfaction and should be considered in future studies.

Fourth, satisfaction was assessed using self-reported measures and may have been influenced by individual expectations, adaptation, and subjective perceptions. Although satisfaction improved over the three-month period, the observational design does not permit attribution of this improvement solely to adaptation, as other patient- or prosthesis-related factors may also have contributed. Fifth, the follow-up period was limited to three months, precluding assessment of whether the observed improvements were sustained over longer periods. Additionally, the questionnaire was developed specifically for this study; although it demonstrated excellent internal consistency, further validation against established patient-reported outcome measures would strengthen its applicability. Future multicenter studies with longer follow-up and larger, more diverse populations are warranted. Further research should also examine the influence of denture design, residual ridge characteristics, Kennedy classification, previous denture experience, patient expectations, socioeconomic factors, and clinician-related factors on satisfaction [17].

## Conclusions

Patient satisfaction improved significantly after three months of removable denture use among both complete and partial denture wearers. Partial denture wearers reported higher satisfaction at prosthesis delivery; however, this between-group difference was no longer statistically significant at three months. The greater improvement observed among complete denture wearers highlights the potential importance of early adaptation and appropriate follow-up. Patient-reported satisfaction provides valuable complementary information for assessing removable prosthodontic treatment outcomes and may help clinicians identify patient concerns and individualize follow-up care.

## Appendices

**Table 8:** Study questionnaire. Response: 1=Very dissatisfied, 2=Dissatisfied, 3=Neutral, 4=Satisfied, 5=Very satisfied. Scoring criteria: 10-17 represented very low satisfaction, 18-25 represented low satisfaction, 26-33 represented moderate satisfaction, 34-41 represented high satisfaction, and 42-50 represented very high satisfaction. Minimum score=10, maximum score=50.

Questionnaire		
Section A. Demographic and patient Information		
No.	Question	Response
D1	What is your age?	____ years
D2	What is your sex?	☐ Male ☐ Female
D3	What is your level of education?	☐ No formal education ☐ Primary ☐ Secondary ☐ Graduate ☐ Postgraduate
D4	What is your occupation?	☐ Employed ☐ Self-employed ☐ Homemaker ☐ Retired ☐ Student ☐ Unemployed ☐ Other: ______
D5	What type of removable prosthesis have you received?	☐ Complete removable denture ☐ Partial removable denture
Section B. Patient satisfaction with removable prosthesis		Response
S1	How satisfied are you with the overall fit and comfort of your denture?	1 ☐, 2 ☐, 3 ☐, 4 ☐, 5 ☐
S2	How satisfied are you with the appearance of the denture and your smile?	1 ☐, 2 ☐, 3 ☐, 4 ☐, 5 ☐
S3	How satisfied are you with your ability to chew and eat food with the denture?	1 ☐, 2 ☐, 3 ☐, 4 ☐, 5 ☐
S4	How satisfied are you with your ability to speak clearly while wearing the denture?	1 ☐, 2 ☐, 3 ☐, 4 ☐, 5 ☐
S5	How satisfied are you with the retention and stability of the denture during daily activities?	1 ☐, 2 ☐, 3 ☐, 4 ☐, 5 ☐
S6	How satisfied are you with the ease of inserting and removing the denture?	1 ☐, 2 ☐, 3 ☐, 4 ☐, 5 ☐
S7	How satisfied are you with the condition and comfort of your gums and oral tissues while wearing the denture?	1 ☐, 2 ☐, 3 ☐, 4 ☐, 5 ☐
S8	How satisfied are you with the ease of cleaning and maintaining your denture?	1 ☐, 2 ☐, 3 ☐, 4 ☐, 5 ☐
S9	How satisfied are you with your ability to wear the denture confidently in social situations?	1 ☐, 2 ☐, 3 ☐, 4 ☐, 5 ☐
S10	Considering all aspects, how satisfied are you with your removable denture overall?	1 ☐, 2 ☐, 3 ☐, 4 ☐, 5 ☐
